# Cholera and Pancreatic Cholera: Is VIP the Common Pathophysiologic Factor?

**DOI:** 10.3390/tropicalmed5030111

**Published:** 2020-07-02

**Authors:** Farzana Afroze, Steven Bloom, Paul Bech, Tahmeed Ahmed, Shafiqul Alam Sarker, John D. Clemens, Farhana Islam, David Nalin

**Affiliations:** 1 International Centre for Diarrheal Disease Research (icddr,b), Dhaka 1212, Bangladesh; farzanaafroz@icddrb.org (F.A.); tahmeed@icddrb.org (T.A.); sasarker@icddrb.org (S.A.S.); jclemens@icddrb.org (J.D.C.); drfarhanaislam@icddrb.org (F.I.); 2 North West London Pathology Consortium, Hammersmith Hospital, Imperial College London, Du Cane Road, London W12 0NN, UK; s.bloom@imperial.ac.uk (S.B.); p.bech@imperial.ac.uk (P.B.); 3 Department of Immunology and Microbial Diseases, Albany Medical College, Albany, NY 12208, USA

**Keywords:** VIP, cholera patients

## Abstract

Background: Cholera remains a major global health problem, causing high output diarrhea leading to severe dehydration and shock in developing countries. We aimed to determine whether vasoactive intestinal polypeptide (VIP), the mediator of pancreatic cholera syndrome, has a role in the pathophysiology of human cholera. Methods: We conducted a prospective observational study of cholera cases hospitalized with severe dehydration. Plasma and stool water levels of VIP were measured just after admission, after complete rehydration (3–4 h), at 24 h post-rehydration and at discharge after diarrhea ceased. Results: In total, 23 cholera patients were examined between January and August 2018. The geometric mean of stool VIP (sVIP) and plasma VIP (pVIP) on admission were 207.67 and 8.34 pmol/L, respectively. pVIP values were all within the normal range (</= 30 pcmol/L); however, sVIP levels were very high at all timepoints, though less so just after rehydration. In multivariable GEE models, after adjustment for covariates, sVIP levels were significantly associated with duration of hospitalization (*p* = 0.026), total stool volume (*p* = 0.023) as well as stool output in the first 24 h (*p* = 0.013). Conclusions: The data suggest that VIP, which is released by intestinal nerves, may play an important role in human choleragenesis, and inhibitors of intestinal VIP merit testing for potential therapeutic benefits.

## Key Point’s/Summary

Cholera patients during profuse watery diarrhea had very high levels of VIP in their stool water, while plasma VIP levels remained normal. This supports the role of VIP in human choleragenesis, as has previously been demonstrated in in vivo animal and in in vitro tissue models.

## 1. Introduction

In 1976, pursuing a possible shared mechanism between cholera and pancreatic cholera syndrome [1,2,3], an abstract described persistent elevated stool vasoactive intestinal polypeptide (sVIP) levels in Bangladeshi cholera patients and in U.S. volunteers contracting cholera or enterotoxigenic *Escherichia coli* diarrhea in vaccine development studies [4]. At admission, cholera patients in shock had elevated plasma VIP (pVIP) levels. These declined to normal levels after correction of shock and dehydration. No VIP was found in the small intestinal luminal fluids of the healthy volunteers. The full report was withheld from publication due to the analyst’s death, with samples having been exhausted. Now, 44 years later, the study has been repeated in cholera patients to determine if the earlier results could be confirmed.

## 2. Background

Cholera patients have elevated intestinal mucosal cyclic amp (cAMP) levels [5], and cholera toxin raises cAMP in in vivo and in vitro animal models and in stripped tissue models [6]. In cats and rats, intraluminal cAMP in denervated intestinal loops also induces luminal secretion [7]. Much prior evidence suggests a role for VIP as a modulator of cAMP levels. VIP, like cholera toxin (CT), enhances tissue cAMP levels and active ion secretion [8]. In cat intestines, intraluminal CT and intra-arterial VIP led to elevated cAMP levels associated with reduced salt and water absorption in villi, but not in crypts, where most secretion into the lumen is believed to originate [9]. However this finding might be due to cAMP turnover being more important in crypt cells than cAMP concentration [10]. Splanchnic nerve stimulation lowers intestinal VIP, thereby reversing VIP-stimulated luminal fluid accumulation [11]. VIP can induce high cAMP levels but can also induce diarrhea without elevating cAMP [9]. The findings in cats linking cAMP, VIP and intestinal fluid accumulation are consistent with a predominant role of reduced unidirectional lumen to plasma sodium and water fluxes found in CT-treated intact in vivo canine jejunal loops (but not in Thiry-Vella loops, in which the plasma to lumen flux was dominant both before and after CT) (D. Nalin and R. Hare, unpublished data). The apparent affinity of VIP for cAMP activation is raised by CT [12] and, in studies of rabbit and human ileal mucosa in vitro, VIP promptly increased cAMP levels, in contrast to no increase after nine other hormones thought to be associated with gut secretion—pentagastrin, glucagon, calcitonin, secretin, carbachol, GIP, serotonin, bradykinin and vasopressin [8]. Substance P affects gut fluid transport by releasing VIP [13]. Luminal 5-hydroxytryptamine induced gut luminal fluid accumulation and its release from enterochromaffin cells was stimulated by CT, but not by the related *E. coli* LT toxin [9,14,15,16]. VIP also has other effects possibly associated with intestinal fluid accumulation, such as raising aquaporin three levels after a 3 h delay [17], similar to the delay between CT exposure and onset of fluid accumulation [18].

While many studies have established that cAMP-mediated changes in net intestinal water and electrolyte secretion is present in cholera, changes in paracellular permeability, such as those caused by the zonula occludens toxin (ZOT) and accessory cholera enterotoxin (ACE) [19], and other possible mechanisms, have been noted [20]. On the other hand, clinical and animal studies of intestinal permeability and vascular flow have not succeeded in identifying such mechanisms in cholera patients [21]. VIPergic pathways actually reduce epithelial paracellular permeability [22].

In vivo studies have the advantage over experimental models like inverted intestinal sacs or biopsied stripped tissues [6,7] of better matching the complete intact pathophysiologic environment by maintaining normal neural and vascular connections. In vivo studies of VIP were conducted in normal human volunteers, in whom intravenously administered VIP induced a decreased absorption of water and electrolytes whilst increasing chloride secretion [23], and induced secretory diarrhea [24,25]. Paradoxically, elevations of cAMP after CT [26] or forskolin [27] are also associated with increased absorption of substrates of the active transport of sodium, such as glucose and glycine, suggesting that all or part of the cAMP elevation (or alterations in cAMP isoform variants) might represent a compensatory mechanism, aimed at overcoming the absorptive defect exemplified by the failure of absorption of plain saline solutions seen in cholera patients [28]. The mechanisms by which cAMP and VIP produce their effects are highly complex and beyond the scope of this report, but have been detailed in recent publications [22,29].

The growing body of evidence relating CT to VIP and gut fluid accumulation leading to diarrhea led us to repeat the earlier unpublished study to confirm a possible VIP role in human cholera pathogenesis. Since diarrhea can be caused by raising VIP levels in either plasma, as in pancreatic cholera syndrome, or by release from nerve endings in the intestinal mucosa, we measured VIP levels in both plasma and cholera rice-water stool water.

## 3. Study Design and Settings

Per protocol, four plasma and concurrent rectal catheter stool sampling points were chosen: the first, following just after correction of shock with intravenous rehydration, but before complete rehydration; the second when rehydration was complete (targeted at 4 h after admission); the third at 28 h, signifying 24 h after completing rehydration, during ongoing maintenance therapy replacing continuing diarrhea; the fourth in convalescence when diarrhea had stopped, though some patients’ stools were still soft, using the findings for comparison with the same patient’s acute phase data. Based on data from the earlier unpublished study, it was anticipated that plasma levels in specimens obtained during or shortly after correcting shock with intravenous hydration would show elevations of pVIP attributable to dehydration and/or shock itself [30]. The rapid return to stable normal pVIP values seen after correcting shock with intravenous rehydration in the earlier study was anticipated as a likely event, for which, the second and third plasma specimens were considered potentially useful in evaluating whether pVIP in cholera patients remained elevated after rehydration during ongoing diarrhea. A return to normal pVIP levels along with continued elevation of sVIP after rehydration during ongoing diarrhea would suggest a local mucosal neuronal VIP source rather than a systemic plasma source, such as seen in pancreatic cholera syndrome. Stool volumes and diarrhea rates were monitored in anticipation of possible correlations with sVIP levels over the course of illness. Diarrhea volumes and rates in cholera are well documented to follow a pattern of steady decline over time, when patients maintain normal hydration [31], and even more strikingly when adjunct appropriate antibiotics are given [32].

## 4. Study Population and Site

The study was conducted at the Dhaka Hospital of the icddr,b (International Centre for Diarrhoeal Disease Research, Bangladesh) between January and August, 2018. The study was approved by the Research Review Committee and the Ethical Review Committee of the icddr,b (Project identification code: PR#17008). Written informed consent was obtained from the participant or caregiver of each participant before enrollment. Patients aged 18–64 years were included if hospitalized with severe dehydration (with absent or impalpable peripheral pulses and lethargic or obtunded mental status) due to acute rice-water diarrhea of <24 h duration, with diagnosis later confirmed by rectal swab culture positive for Vibrio *cholerae* 01 or 0139. Patients with preadmission antibiotic therapy or complicating comorbidity were excluded.

On admission, patients’ respiratory and circulatory statuses were assessed, and intravenous rehydration was started within ninety seconds. Patients received an initial bolus of 30 mL/kg of isotonic fluid (normal saline or acetate solution) [33] over 30 min, followed by 70 mL/kg over the next 2.5–3.5 h, to replace fluid deficit equivalent to ≥10% body weight within 3–4 h. Patients were kept on cholera cots and ongoing stool volumes collected into calibrated buckets were monitored q2h and recorded q4h. Urine output was collected and measured separately. After initial rehydration, ongoing losses were matched with isotonic fluid (normal saline or acetate) for the first 24 h, after which, ongoing stool losses were replaced with oral rehydration solution [34,35]. Single dose oral azithromycin (1 g) was administered after intravenous rehydration with abatement of vomiting, based on a local antibiogram.

Parameters observed included diarrhea duration and volume, plasma parameters of hydration and electrolyte status and glucose to monitor diarrhea-associated hemoconcentration, bicarbonate loss and associated acidosis and potassium abnormalities, and hypoglycemia. As noted above, VIP levels in both plasma and diarrhea stool water were monitored to detect any elevations originating either systemically or produced by neurons in the intestinal mucosa.

## 5. Stool and Blood Samples for VIP Assay

We obtained stool and blood specimens at the following timepoints: just after enrolment into the study and initial stabilization of patients (time 0); 3–4 h (after full rehydration with replacement of fluid deficit equivalent to ≥10% body weight); 24 h after complete rehydration, during ongoing diarrhea in the presence of normal vital signs; at time of discharge.

The 30 mL rectal catheter specimens of rice-water stool were obtained and placed immediately into tubes containing 0.15 TIU of chilled aprotinin/mL of stool water (Trasylol, Sigma-Aldrich, Activity: 3–8 TIU/mg solid). Pre-discharge, freshly passed non-liquid stool specimens were treated with chilled distilled water (1:3 dilution) in a tube containing chilled aprotinin equivalent to 2% of the total volume of stool plus distilled water. Stool samples were cold centrifuged at 13,523 g, and the supernatant was retrieved in a disposable syringe to filter through a 0.2 micron Millipore filter (Whatman 25 mm GD/X syringe filter, Sigma-Aldrich). Small volumes of filtered supernatant from the enrolment samples were re-cultured to ensure removal of vibrios. Venous blood samples were collected and cold centrifuged in lithium heparin tubes containing 0.15 TIU of chilled aprotinin/mL blood. We processed all specimens within 15 min after collection and kept them frozen (−80 °C) until they were shipped in dry ice to the UK for RIA assay [36]. We also collected 5 mL blood to measure electrolytes, plasma specific gravity, glucose and blood urea nitrogen.

## 6. Statistics

To plot the distribution of stool and plasma VIP values after natural log transformation on admission, rehydration completion, 24 h post-rehydration and discharge, we used dot plots. The stool water and plasma VIP levels did not follow a normal distribution as depicted by box and whisker plots, Q–Q plots (quantile–quantile plots) and Shapiro–Wilk tests, but followed normal distribution after log transformation. Therefore, we reported mean as geometric mean (GM) with a 95% confidence interval. We applied generalized estimating equation (GEE) models with an exchangeable correlation and Gaussian family structure to compare longitudinal variations in stool water and plasma VIP concentrations at four different timepoints for each patient, while adjusting for possible intra-subject correlations. Application of ordinary regression analysis may confer biased results for repeated measures data, whereas GEE methodology indicates how the mean of an outcome variable of a participant changes with covariates while adjusting intra-subject correlations related to repeated measures outcome data [37]. GEE is the best model for repeated measures and gives more robust results than ANOVA. In the GEE model, stool water VIP was the outcome variable, while independent variables were both time-varying covariates as well as non-time-varying covariates. As we included log transformed variables in the GEE model, so as to interpret our results, we applied the exponential (inverse of a log) function in the GEE models. In multivariable models, potential covariates with *p* < 0.2 in the bivariate analysis were included. A *p*-value < 0.05 was considered statistically significant. We analyzed data using Stata version 13.1 (Statacorp LP, College Station USA).

## 7. Results

We included 23 cholera cases for analysis; stool specimens of twenty-two (96%) cases grew Vibrio *cholerae* O1 biotype El Tor serotype and one (4%) *V. cholerae* 0139 biotype. Patient characteristics are shown in Table 1.

**Figure 1:**
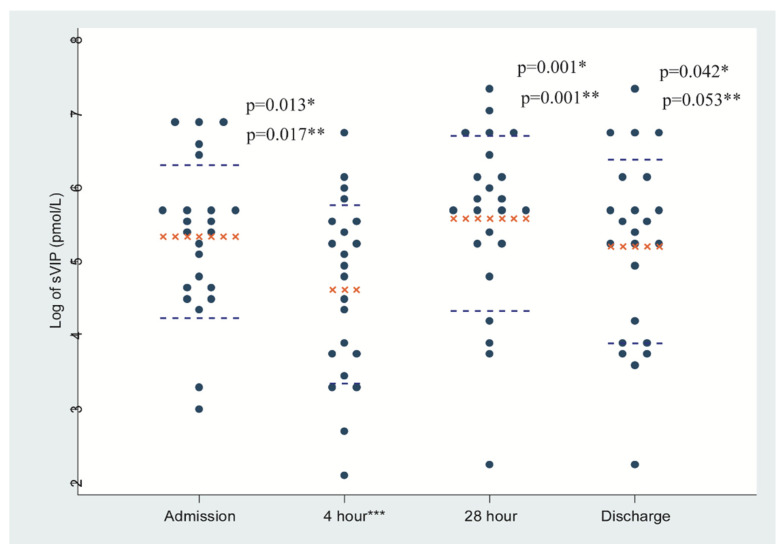
Distribution of average values of stool VIP after log transformation. Dot plot: Stool VIP concentrations are presented separately for admission, 4 h, 28 h and discharge samples. Bar representing mean ± SD. * *p* values derived from the bivariable GEE models. ** *p* values derived from the multivariable GEE models. *** 4 h time point is the reference category.

sVIP and pVIP concentrations and sVIP distribution are shown in Table 2 and Figure 1. The geometric means (GM) of sVIP and pVIP on admission were 207.67 and 8.34 pmol/L, respectively (Table 2). pVIP values were all within the normal range (</= 30 pcmol/L), though pVIP levels after partial rehydration were significantly higher than post-rehydration levels (*p* = 0.001).

In both bivariable and multivariable models, sVIP concentration after complete rehydration at the 4 h timepoint (replacement of fluid deficit equivalent to ≥10% body weight) was considered as the reference category. As some patients reached complete rehydration by 3 h post-admission, the 4 h timepoint represents a range of 3–4 h post-admission. In addition, some patients’ diarrhea ceased by 24 h post-rehydration, so those values were considered as discharge values in the analytic models. In fact, the geometric means were virtually identical whether using data from patients contributing four specimen sets or from those whose diarrhea terminated at 24 h post-rehydration. At all timepoints, sVIP levels were far higher than pVIP levels, though unadjusted models revealed that the 4 h sVIP level was lower than at the other timepoints (Figure 1).

In multivariable (adjusted) GEE models, after adjusting other covariates, the comparison of discharge sVIP levels with 4 h levels did not reach significance. In multivariable models, after adjustment for covariates, sVIP levels were significantly associated with duration of hospitalization, total stool volume as well as stool output in the first 24 h (Table 3).

Net sVIP losses in cholera diarrhea were estimated based on ten patients with complete specimen sets and based on diarrhea output most closely matched in time with the time of specimen collection. For the admission timepoint, it was assumed that a mean of 1.0 L of diarrhea fluid remained unexpelled in the intestine on admission. The values at 0, 4, 28 and discharge timepoints were, respectively (pcg, mean ± s.d.): 308 ± 267, 422 ± 244, 749 ± 1126 and 188 ± 225.

## 8. Discussion

This is the first full prospective report of plasma and stool VIP levels in cholera patients. The findings confirm that in cholera patients, sVIP levels are elevated. The findings are consistent with earlier in vivo animal and in vitro tissue studies suggesting a neural mechanism of pathogenesis, though those studies did not measure sVIP. The current study shows a clear link bridging prior work to stool VIP in cholera patients. This underlines the possibility of new treatments aimed at interrupting the diarrheagenic process and shortening disease duration more than what is possible with antibiotics and fluid replacement alone.

The slightly higher pVIP levels, which fell after rehydration and correction of shock, probably represent a residual of dehydration and the recent correction of shock [30]. In any case, all pVIP values were within the normal range, ruling out any systemic pVIP elevation during cholera diarrhea. In contrast, the persistent high sVIP levels during the course of cholera diarrhea indicate that cholera diarrhea is associated with enhanced intestinal VIP production. The luminal VIP levels may have a direct mucosal effect or may represent the overflow from neural production in the mucosa. Intestinal VIP is neuronally controlled [7,11] and luminal VIP levels are not the product of direct transfer from plasma. In any case, pVIP levels before and after rehydration were within the normal range and were far below sVIP levels, even after rehydration. Slightly elevated blood peptide hormone levels have been reported in patients with presumed infectious or AIDS-related diarrhea [38,39], though stool volumes, diarrhea rates and stool water VIP levels were not reported. The relationship of these slightly high pVIP levels to sVIP levels in cholera patients is unclear, and may possibly have been due to dehydration levels, which were also not reported.

Sorting out the relative importance of VIP per se or in concert with other neuronally generated mediators of intestinal water and salt loss [7,12,13] awaits further study, but the bulk of evidence suggests that VIP may play a dominant role. Further study is also need to determine whether, in cholera patients, VIP’s effects on cAMP activity influence the cAMP-enhanced absorption of actively transported sugars and amino acids [26], and superior absorption of oral rehydration solutions with glucose plus glycine [28] or with rice [40], rich in starch and amino acids [41,42]. This effect is not seen in diarrheal illnesses caused by other pathogens [43], in which VIP and cAMP may play a lesser role.

## 9. Limitations of this Study

While the results clearly confirm that cholera patients have elevated sVIP, the associations with total and 24 h stool volumes and diarrheal duration do not prove a causal relationship. To explore this possibility, the effects of VIP inhibitors or antagonists on cholera toxin-induced intestinal fluid losses in suitable animal models, such as the dog [44] or cat [45] cholera models, are warranted, elevated VIP levels in intestinal venous blood having already been demonstrated in the latter. The presence of sVIP levels exceeding plasma levels in convalescence may represent residual luminal VIP reaching the lower bowel after antibiotic therapy eradicates vibrios, stopping cholera toxin production. The correlations between sVIP and diarrhea duration and volume are notable, but this study was not designed to monitor diarrheal production rate and simultaneous luminal VIP levels in the small intestine. sVIP levels reflect production at the intestinal level at a prior timepoint, depending on intestinal transit time, influenced variably by intestinal motility. Studying these variables would require the intubation technique in cholera studies in human volunteers such as those previously reported in cholera vaccine pilot studies [46].

## 10. Conclusions

This study is the first fully documented report of high sVIP levels in cholera patients. High VIP levels in cholera patients’ stool water may reflect an important role of VIP in the pathophysiology of cholera diarrhea. The findings suggest that human cholera diarrhea may be mediated by heightened intestinal neural production of VIP and luminal release of VIP, consistent with earlier in vitro and in vivo animal model studies suggesting participation of a neural/hormonal mechanism in pathogenesis.

## 11. Authors’ Translational Perspective

While lidocaine and tetrodotoxin given after CT reduce its diarrheagenic effects [47], their neurologic and cardiovascular toxicities preclude use in cholera patients. Studies have demonstrated the effectiveness of somatostatin and methionine-enkephalin in antagonizing VIP’s diarrheagenic activity when delivered intra-arterially [48], but the intestinal intra-arterial route is not clinically feasible, and intravenous administration of somatostatin had no effect on stool output in cholera patients [49]. Other VIP antagonists have also shown activity when administered I.V. immediately after cholera toxin [50,51], but the applicability of this to the clinical situation, when patients are seen long after diarrhea has been established, is not known. The identification of highly potent somatostatin-receptor agonists capable of inhibiting secretion after luminal mucosal surface application [52] suggests that these agents merit studies in appropriate animal cholera models such as the dog or cat to evaluate their possible suitability for human trials. It is striking that despite several decades of research pointing to a VIP role in choleragenesis, no translational study has appeared demonstrating a clinical benefit of antagonists or other compounds with antihormonal activity. Hopefully, the current confirmation of a VIP role in cholera patients may reawaken interest in such studies, including testing newer highly potent and mucosally active compounds to determine if they can safely interrupt the diarrheagenic process and shorten disease duration more than what is possible with antibiotics and fluid replacement alone.

## Figures and Tables

**Table 1:** Clinical and laboratory characteristics of cholera patients with severe dehydration/hypovolemic shock in Dhaka, Bangladesh.

Characteristics	Total (*n* = 23)
Age (in years)	33 ± 10
Male (n, %)	20/23 (87)
Diarrhea duration (h)	41 ± 19
Emesis duration (h)	2 (1,3)
Abdominal pain (n, %)	4/23 (17)
Total stool volume (mL/kg)	141 (109, 191)
Urine output (mL/h)	136 ± 67
Stool output in 1st 24 h (mL/day)	8000 (5500, 10,500)
Total intravenous fluid (mL/kg/h)	11 (8.4, 12.75)
Duration of hospital stay (h)	63 ± 23
Admission blood glucose level (mmol/L)	8 (6,10) (4.2–7.8)
Admission plasma sp.gr.	1.0480 ± 0.006 (1.0232–1.0279)
Admission serum.Na^+^ (mmol/L)	133.28 ± 2.30 (135–146)
Admission serum. K^+^ (mmol/L)	5.16 ± 1.15 (3.5–5.3)
Admission serum. Cl^−^ (mmol/L)	102.26 ±3.06 (97–106)
Admission serum TCO_2_ (mmol/L)	18.14 ± 4.63 (23–30)
Anion gap (mmol/L)	17.95 ±3.82 (7–21)
BUN (mmol/L)	14.80 ±4.80 (5–20)
Urine Sp. gr. at 4 h *	1.04 ± 0.005 (1.005–1.030)
Serum Na+ (mmol/L) at 4 h *	135 ± 2.76
TCO2 (mmol/L) at 4 h *	22.5 ± 3.57
Blood glucose level (mmol/L) at 4 h *	5.4 (4.88, 7.39)
Mean stool VIP (pmol/L)	307 (168,410)
Peak stool VIP (pmol/L)	591 (283,855)
*Vibrio cholerae* 01, El Tor	22/23 (96)
*Vibrio cholerae* 0139	1/23 (4)
*Vibrio cholerae* 01, El Tor Ogawa	17/22 (74)
*Vibrio cholerae* 01, El Tor Inaba	5/22 (22)

Data are presented as n (%), mean ± SD, or median (IQR). * Measured after complete rehydration. Abbreviations: VIP—vasoactive intestinal polypeptide; Total intravenous fluid (mL/kg/h)—replacement of fluid deficit equivalent to ≥10% body weight and ongoing loss. Normal reference values in brackets.

**Table 2:** Variations of stool and plasma VIP concentrations (pmol/L) over four timepoints before and after rehydration among cholera patients.

VIP Measurement Time Points	Stool VIP (pmol/L)		Plasma VIP (pmol/L)	
	Geometric Mean *	95% CI	Geometric Mean *	95% CI
Admission (0 h)	207.67	132.74, 324.90	8.34	5.53, 12.59
At 4 h (after complete rehydration)	101.55	59.40, 173.60	3.84	2.59, 5.67
At 28 h	265.97	159.17, 444.41	2.67	1.82, 3.92
At discharge	181.40	105.17, 310.63	3.31	2.26, 4.83

* Mean of stool and plasma VIP concentrations after log transformation.

**Table 3:** Associations of stool water VIP (pmol/L) in patients with cholera and severe dehydration/hypovolemic shock at four different timepoints before and after rehydration using generalized estimating equations models.

Characteristics		Unadjusted exp (Coefficient) * with 95% CI	*p*-Value	Adjusted exp (Coefficient) * with 95% CI	*p*-Value
Duration of hospital stay (h)		1.01 (0.992, 1.022)	0.161	1.01 (1.001, 1.023)	0.026
Total stool volume (mL/kg)		1.00 (0.993, 1.000)	0.087	1.01 (1.002, 1.025)	0.023
Stool output in first 24 h (mL)		1.00 (0.999, 1.000)	0.075	1.00 (0.999, 0.999)	0.013
Total IVF (mL/kg/h)		0.93 (0.864, 1.007)	0.077	0.94 (0.875, 1.021)	0.157
Stool VIP (pmol/L) measurement time points	4 h	ref		ref	
	Admission	2.01 (1.174, 3.762)	0.013	2.03 (1.136, 3.640)	0.017
	28 h	2.69 (1.504, 4.818)	0.001	2.60 (1.455, 4.662)	0.001
	Discharge	1.83 (1.026, 3.287)	0.042	1.77 (0.992, 3.179)	0.053

* Ratio of geometric mean: ratio of admission/28 h/discharge value to reference value.
